# Timing, Tools, and Thinking: H5P-Driven Engagement in Flipped Veterinary Education

**DOI:** 10.3390/vetsci12101013

**Published:** 2025-10-20

**Authors:** Nieves Martín-Alguacil, Rubén Mota-Blanco, Luis Avedillo, Mercedes Marañón-Almendros, Miguel Gallego-Agundez

**Affiliations:** Departmental Section of Anatomy and Embryology, School of Veterinary Medicine, Universidad Complutense de Madrid, 28040 Madrid, Spain; rubenmot@ucm.es (R.M.-B.); luiavedi@ucm.es (L.A.); mdemaran@ucm.es (M.M.-A.); migugallt@ucm.es (M.G.-A.)

**Keywords:** cognitive engagement, continuous assessment, constructivist pedagogy, digital learning analytics, flipped learning, H5P^®^ interactive modules, Marzano taxonomy, student engagement

## Abstract

Traditional lectures in veterinary anatomy often limit student engagement and higher-order thinking. The flipped classroom (FC) model shifts foundational content to independent study using interactive tools such as H5P^®^ and Wooclap^®^, reserving classroom time for collaborative problem-solving. Objective: To evaluate the impact of the FC model on student engagement, preparation habits, and cognitive performance in veterinary anatomy, focusing on the respiratory and cardiovascular systems. Methodology: The intervention was implemented over two academic years (2023/24 and 2024/25) and included continuous assessment, cognitive-level evaluations based on Marzano’s taxonomy, platform analytics, and anonymous student surveys. Results: Platform data showed high engagement, with completion rates exceeding 90%. Students who prepared 2–3 days in advance performed better on application and integration tasks. Survey responses indicated a shift from passive video viewing to active learning strategies, such as structured note-taking and strategic time management. By 2024/25, 85% of students dedicated 30+ min to preparation, compared to 48% the previous year. Conclusion: The FC model fostered autonomy, spatial reasoning, and clinical contextualization. Aligned with constructivist principles, it supported Intended Learning Outcomes through adaptive scaffolding. Despite institutional challenges, the model proved scalable and pedagogically coherent, warranting further longitudinal research and broader curricular integration.

## Simple Summary

This study examined whether the flipped classroom (FC) model could enhance learning in veterinary anatomy, with a particular focus on the respiratory and cardiovascular systems. Rather than attending traditional lectures, students studied the core content independently using interactive videos and quizzes and then used the class time for problem-solving and anatomical discussions. The research, which included digital engagement data, student performance on cognitive-level tasks and survey feedback, was conducted over two academic years. The results showed that students who prepared earlier, especially 2–3 days before class, performed better on complex questions. Survey responses revealed a shift from passive habits, such as watching videos once, to active strategies, such as note-taking and deeper study. The flipped model helped students to develop autonomy, critical thinking and spatial reasoning skills. It also aligned well with educational frameworks and supported key learning outcomes. However, challenges such as limited staffing and resistance to change were also identified. In conclusion, when well-designed and supported, flipped teaching can be a powerful tool for veterinary education.

## 1. Introduction

In higher education, there has been a growing shift away from passive learning approaches, such as traditional lectures, towards active learning strategies that promote student engagement and deeper cognitive involvement [1,2,3]. Multiple studies demonstrate that active learning approaches consistently improve knowledge acquisition, skill development, engagement, motivation, and knowledge retention compared to passive methods [1,2,3]. In recent years, flipped classroom (FC) models have become more prominent in health sciences education due to their potential to encourage autonomy, engagement and deeper cognitive processing [4]. However, the implementation of these models in veterinary anatomy has received limited attention, particularly regarding the influence of specific instructional strategies on student behavior and learning outcomes. This study aims to address this gap by examining the impact of continuous assessment and interactive digital tools (H5P^®^, Wooclap^®^) on student engagement over two academic years. Specifically, we will investigate the following: (1) How changes in instructional design affect attendance and preparation habits; (2) Whether preparation timing influences performance at different cognitive levels; (3) How procrastination evolves in response to evaluative reinforcement; (4) Whether the integration of interactive platforms supports higher-order thinking. Through the analysis of behavioral and performance data, this study seeks to clarify how flipped methodologies can be optimized for veterinary anatomy education.

Teaching veterinary anatomy to early-stage students presents a dual challenge: fostering an understanding of complex physiological systems and developing spatial and procedural competence. This is particularly evident in the study of the respiratory and cardiovascular systems, where students must integrate theoretical knowledge with identifying anatomical structures and their functional relationships. Traditional lecture-based approaches often fail to meaningfully engage first-year students, particularly when confronted with abstract content and limited prior experience of anatomical reasoning [5,6]. In recent years, however, the FC model has emerged as a promising pedagogical strategy to address these challenges [7]. The FC in which students interact with content independently before class and apply their understanding collaboratively during in-person sessions, has gained particular traction [4], as well as, in anatomy learning [8]. By shifting the acquisition of foundational knowledge to pre-class activities and dedicating in-class time to active learning, the FC model promotes deeper engagement, peer collaboration and the development of higher-order thinking skills [4,8,9]. This model has gained traction in medical and health sciences education, and is increasingly represented in veterinary literature [4,8,9]. This approach encourages students to engage with core content independently before class, thereby maximizing in-person sessions for applied learning, peer interaction and instructor-led clarification [10,11]. This method is effective because it fosters student autonomy, deepens conceptual understanding and enhances academic performance, particularly in subjects requiring strong foundational knowledge and spatial reasoning, such as veterinary anatomy [11]. Within this pedagogical framework, the integration of digital tools has become increasingly relevant [9].

Understanding how students engage with digital learning resources provides valuable insights into the effectiveness of instructional design [4,12]. Understanding student engagement as the emotional, behavioral, and cognitive involvement a student exhibits during the learning process [12]. Metrics such as video visualization frequency, time spent on pre-class preparation and classroom attendance reveal behavioral trends and underlying study habits and cognitive engagement. In time-constrained curricula, optimizing learning efficiency depends on aligning the time students spend preparing with the cognitive demands of the course materials. This remains a challenge in FC models, where it is crucial to ensure that students have sufficient time and resources for meaningful engagement before class [13,14].

In the FC model, the time students dedicate to reviewing materials before face-to-face sessions directly affects the depth and quality of their engagement in class [4,15]. In flipped and blended learning environments, the connection between pre-session content and in-class activities is a cornerstone of effective instructional design. When students do not adequately engage with preparatory materials—or when those materials are poorly aligned with classroom tasks—the resulting gap can hinder knowledge construction and reduce learning efficacy [16].

Procrastination is a well-documented behavioral challenge in higher education, particularly in self-directed learning environments such as the FC model [17,18]. Although FC methodologies promote autonomy and flexibility, they also require students to manage their time effectively and engage with preparatory materials in advance. In anatomy education, where the cognitive demands are high and the complexity of the content varies, procrastination can undermine the benefits of active learning by limiting students’ readiness for in-class tasks. Therefore, understanding how procrastination manifests and evolves within flipped settings is crucial for designing interventions that encourage timely engagement and deeper cognitive processing.

When pre-session resources, such as interactive H5P videos, are designed efficiently and clearly and are aligned with cognitive goals, they maximize the impact of limited preparation time [14]. This is particularly important in intensive curricula, where students must balance their workload with developing a deep conceptual understanding. Effective pre-class preparation enables learners to participate actively in problem-solving and collaboration. However, if the materials are poorly structured or overly time-consuming, students may either underprepare or rely on passive review strategies, which limit the effectiveness of classroom activities. Conversely, when digital content is concise, engaging and tailored to support specific learning objectives, it promotes timely revision and cognitive priming, making the most of in-person instruction [19]. Ultimately, aligning the design of pre-session materials with realistic student time constraints ensures that independent study is both manageable and meaningful, creating a strong foundation for successful face-to-face learning experiences. By tracking video visualization, educators can determine which content is revisited, which segments are skipped and how frequently learners interact with materials outside the classroom. A high frequency of views may indicate high motivation, or that the content is complex and requires deeper review. Time spent preparing, particularly when measured across specific intervals before class (e.g., one day versus several days prior), offers clues about self-regulated learning and procrastination patterns. When paired with performance data, this metric can show whether earlier engagement translates into better comprehension and retention outcomes. Although traditional, classroom attendance still holds diagnostic value. Consistent attendance often indicates student accountability and active participation. When these metrics are considered together, educators can assess whether their instructional model—such as flipped learning or blended formats—effectively promotes autonomy, reflection, and collaboration. Leveraging these data points enables educators to refine their teaching strategies, adapt their pace and design interventions that better support diverse learning needs in technology-enhanced environments.

The FC intervention implemented in this study aimed to foster five core competencies that are essential for first-year veterinary students: anatomical identification; spatial and functional integration; clinical application; structured study habits; and collaborative reasoning. These objectives informed the instructional design, combining digital pre-class materials with team-based in-class activities, and guided the evaluation framework across two academic years (2023/24 and 2024/25). The study aimed to assess the relationship between students’ independent study time and their learning outcomes, paying particular attention to attendance patterns, content access behaviors and student feedback collected through integrated digital platforms. By comparing cohorts across both years, the study also examined how changes in evaluation models, such as the introduction of continuous assessment, may influence engagement and performance. The intervention was structured around specific Intended Learning Outcomes (ILOs), cognitive exercises mapped to Bloom’s Taxonomy and behavioral indicators tracked via platforms such as H5P^®^ and Wooclap^®^, which supported formative assessment and learner feedback [20,21].

## 2. Materials and Methods

### 2.1. Study Design and Context

This study used a comparative observational design to evaluate the impact of FC methodologies on student engagement and performance in veterinary anatomy education. The analysis focused on two consecutive academic years (2023/24 and 2024/25) at the Faculty of Veterinary Medicine at the Universidad Complutense de Madrid. Both cohorts participated in the same anatomy course, but there were key differences in the instructional design: the 2023/24 cohort experienced a non-evaluative FC model, whereas the 2024/25 cohort engaged with a revised version that incorporated continuous assessment and graded in-class activities. The study aimed to explore how these pedagogical adjustments influenced attendance, preparation habits, procrastination behavior and cognitive performance across Marzano’s taxonomy levels [22].

### 2.2. Participants and Cohorts

This study was conducted with first-year veterinary medical students enrolled in a foundational Anatomy course during the 2023/24 and 2024/25 academic years. A total of 235 students participated in the first year of implementation, followed by 208 students in the second year. All of the students involved in the study had comparable academic backgrounds and were given the same access to course materials, including lecture slides, textbooks and institutional learning platforms. Before the intervention began, participants completed an initial survey designed to gather information about their educational background and study habits. The data collected helped to validate the group allocation process. To encourage meaningful peer interaction, some discussion groups comprised only students who were retaking the course. This arrangement aimed to encourage equal participation among individuals with similar academic backgrounds and knowledge of the course content. While most subjects followed a traditional expository lecture format throughout the semester, the anatomy course adopted a flipped learning approach during its face-to-face theoretical class sessions. Students were required to engage with preparatory materials—specifically, H5P^®^ interactive videos—prior to attending theorical class. These videos were designed to deliver core anatomical content in an engaging, self-paced format, enabling students to prepare for in-class activities more effectively.

### 2.3. Instructional Framework

The FC model was implemented in both academic years, following a consistent structure. Students were expected to engage with preparatory materials before class, participate in active learning sessions during class and consolidate their learning through activities after class. In the 2023/24 academic year, the FC approach was purely formative, with no graded in-class activities. Students accessed asynchronous video content and interactive resources but were not formally evaluated during sessions. By contrast, the 2024/25 cohort experienced a revised FC model that incorporated continuous assessment through graded in-class tasks aligned with Marzano’s taxonomy. These tasks were designed to promote higher-order thinking and strategic engagement. While the instructional sequence remained consistent, the evaluative component introduced in the second year aimed to reinforce accountability, reduce procrastination and encourage deeper cognitive processing.

To assess the effectiveness of the FC intervention in theoretical instruction, we collected and analyzed a combination of platform engagement data, formative assessment results, and student feedback. The goal was to evaluate how each pedagogical strategy supported the achievement of learning objectives and student engagement across two academic years. The study aimed to analyze how students engaged with independent study materials during a flipped learning experience and the effect this had on their learning outcomes. Platform interaction data and self-reported behaviors were used as indicators of preparation and study habits. The FC methodology [10,11] was implemented across both academic years. Students engaged with content independently before class and then applied knowledge during guided face-to-face sessions.

The instructional design of the FC intervention was informed by Marzano’s framework for effective teaching [22]. Pre-class materials were developed using H5P^®^ and included narrated videos, interactive labeling exercises and embedded formative assessments. Each module was aligned with specific learning objectives, such as identifying structures in the thoracic cavity and understanding their functional relationships. In-class sessions were structured to encourage collaborative discussion and the application of prior learning through clinical scenarios and anatomical reasoning tasks. The design incorporated Marzano’s strategies, such as setting objectives, providing feedback and using non-linguistic representations, to support cognitive engagement and self-regulated learning. The FC’s instructional design was deliberately structured to support the achievement of targeted learning objectives within the first-year veterinary anatomy curriculum, with a specific focus on the respiratory and cardiovascular systems. These objectives were derived from the official syllabus and refined to reflect the cognitive and procedural competencies expected at this stage of training.

### 2.4. Digital Tools and Resources

Digital tools (ICT): H5P^®^ and Wooclap^®^ were used on the Moodle virtual campus platform to support independent study and formative assessment in veterinary anatomy, aligned with specific learning objectives. Digital platforms (H5P^®^ and Wooclap^®^) were used to monitor and assess student engagement and study habits.

The FC intervention was designed to help first-year veterinary students master the anatomy of the respiratory and cardiovascular systems. It followed a two-phase model: autonomous pre-class preparation using digital resources; and in-class collaborative learning focused on application, discussion and anatomical identification.

Pre-class material: Students accessed curated digital content in the form of H5P^®^ interactive videos via the institution’s Moodle-based learning platform. These materials were designed to introduce core concepts and terminology, enabling students to familiarize themselves with the theoretical foundations prior to attending class. The interactive H5P^®^ modules included quizzes to reinforce spatial understanding. All materials were aligned with specific ILOs and students were required to complete the embedded formative assessments before the in-class session.

In-class activities: Classroom sessions were structured around team-based learning principles, with students working in small groups to solve anatomical challenges and apply their pre-class knowledge through cognitive exercises and formative assessments using Wooclap^®^ tool or hand-writing exercises.

### 2.5. Assessment and Evaluation Criteria

This subsection outlines the formative and summative assessment strategies used during the intervention. Each cognitive exercise was designed to reinforce specific anatomical and clinical competencies while promoting active engagement. Table 1 summarizes the types of activities and their pedagogical roles.

Table 1 summarizes the assessment activities integrated into the FC intervention, outlining their respective pedagogical roles. Each activity was selected to reinforce specific cognitive and practical competencies that are aligned with the learning objectives and ILOs of the course. Real-time polling using platforms such as Wooclap^®^ provided instructors with immediate feedback and allowed them to identify misconceptions and adjust their teaching approach accordingly. Problem-based anatomical reasoning tasks encouraged students to apply their knowledge to realistic diagnostic scenarios, thereby fostering clinical relevance and a deeper understanding of anatomy. The integration of theoretical and practical components within each session supported conceptual mastery of topics such as blood flow dynamics and respiratory mechanics, as well as hands-on skills such as identifying anatomical landmarks. Together, these activities contributed to a comprehensive evaluation framework that captured student engagement, performance and progression across cognitive levels.

### 2.6. Learning Objectives

The intervention was designed to foster five core competencies essential for first-year veterinary students. These objectives guided both the instructional design and the evaluation framework.

Table 2 outlines the five core learning objectives that informed the design of the FC intervention. These objectives were chosen to reinforce foundational anatomical knowledge and encourage higher-order thinking and collaboration. Each objective played a distinct role in guiding students through the cognitive progression from recognition to application. For instance, identifying thoracic structures provided a foundation for grasping spatial relationships and functional integration, thereby supporting clinical reasoning. Structured study habits were encouraged through pre-class preparation, while collaborative reasoning was fostered during in-class, team-based activities. Together, these objectives ensured that the intervention addressed both individual and group learning processes, thus aligning with the course’s intended outcomes and assessment strategy.

### 2.7. ILOs

The ILOs were aligned with the course’s anatomical and clinical goals. They served as benchmarks for both instructional delivery and performance evaluation.

Table 3 shows the Intended Learning Outcomes (ILOs) that were prioritized in the FC intervention. These outcomes were carefully selected to align with the anatomical and clinical demands of the first year of veterinary education, with a particular focus on the thoracic region. Each ILO addresses a distinct level of cognitive and practical competence, progressing from basic recognition to applied reasoning and collaborative problem solving.

ILO 1 focuses on accurately identifying and describing major thoracic structures in domestic mammals. This includes not only isolated anatomical elements, such as the heart, lungs, trachea and bronchi, but also their spatial organization and functional relationships. Mastery of this outcome enables students to understand the heart’s position within the mediastinum, the bronchi’s branching in relation to pulmonary vessels, and the continuity between the pleural cavities and the thoracic wall. Such topographical knowledge is vital for interpreting diagnostic images, planning surgical approaches and avoiding iatrogenic injury during clinical procedures.

ILO2 builds on this anatomical foundation by emphasizing the functional interactions between respiratory and cardiovascular components. Students learn to integrate concepts such as circulation, ventilation and gas exchange, which are critical for understanding physiological dynamics and pathophysiological disruptions. This outcome further reinforces the anatomical context by linking structure to function—for example, demonstrating how the proximity of the pulmonary arteries to the bronchi affects airflow and perfusion.

ILO3 introduces students to applied clinical reasoning. By interpreting basic scenarios involving cardiopulmonary dysfunction, learners begin to link anatomical knowledge with clinical signs, imaging results and therapeutic decision-making. This is a particularly relevant outcome for surgical training, as it requires students to visualize internal structures in three dimensions and anticipate anatomical variations or pathological changes.

ILO4 addresses the collaborative dimension of anatomical learning. Through small-group problem solving and structured communication, students refine their ability to describe anatomical relationships using precise terminology and visual references. This outcome fosters peer learning and prepares students for interdisciplinary teamwork in clinical settings.

Together, these ILOs facilitate a comprehensive understanding of thoracic anatomy that goes beyond mere memorization. They cultivate spatial reasoning, functional integration and clinical applicability—skills that are indispensable for safe and effective veterinary practice.

### 2.8. Behavioral Indicators of Data Collection

This subsection describes the timeline and methods used to collect behavioral and performance data. Each instructional sequence was designed to elicit measurable engagement and cognitive progression.

Table 4 summarizes the behavioral indicators and data collection tools used to evaluate student engagement and performance throughout the FC intervention. These indicators were chosen to capture observable behaviors and measurable outcomes in the pre-, in-, and post-class phases of instruction.

Qualitative insights into students’ perceptions, expectations, and self-reported engagement were provided by baseline and post-intervention surveys. These responses were thematically coded to identify patterns in motivation, preparation habits, and the perceived usefulness of the flipped format.

Access metrics from the H5P^®^ platform, including the number of views, access timing and completion rates, served as indirect indicators of study behavior. While these metrics do not directly measure attention or comprehension, they provide valuable data on how students interact with digital content and manage their preparation time. The five-day access window prior to each session was intentionally chosen to encourage early engagement and discourage last-minute preparation.

Segmenting videos into two 10–15 min modules per topic supported cognitive load management and facilitated notetaking. This structure was designed to align with multimedia learning principles and optimize retention.

Wooclap^®^ was used during in-class sessions to monitor attendance, deliver formative assessments and collect real-time feedback. Spot polls and interactive exercises were aligned with the Intended Learning Outcomes and cognitive levels, enabling instructors to assess understanding and adapt their teaching approach as necessary.

The cognitive exercises were designed to span the four levels of Bloom’s taxonomy: recall, interpretation, application and integration [23]. During the 2023/24 academic year, active learning scores contributed towards final exam grades. The following year, continuous assessment was introduced, and formative tasks became part of the final course evaluation. This shift enabled a more nuanced analysis of the relationship between engagement patterns and performance.

Class attendance, video visualization timing, and note-taking behaviors were treated as observational variables, while student performance scores across the four cognitive taxonomic levels constituted the only quantified data subjected to statistical analysis. These indicators were analyzed using statistical and thematic methods to explore correlations between engagement patterns and learning outcomes.

### 2.9. Statistical Analysis

Students’ performance in solving exercises at the four cognitive taxonomic levels (scored 0–10) was subjected to statistical analysis. Normality of the data was tested using the Shapiro–Wilk test, which revealed non-normal distributions. Consequently, comparisons across the four student groups were performed using the non-parametric Kruskal–Wallis test, followed by Dunn’s post hoc test for pairwise comparisons. A significance level of *p* < 0.05 was applied. Other variables, such as class attendance and video visualization across different years, were considered descriptive/observational and were therefore not analyzed with inferential statistics. All analyses were conducted using STATA version 17 (StataCorp, College Station, TX, USA).

## 3. Results

### 3.1. Platform Analytics

Student engagement with the FC materials was monitored through the institutional virtual learning environment, which provided detailed usage metrics. These analytics offered insight into how students interacted with the digital content prior to in-class sessions. Across both academic years, students demonstrated consistent engagement with the preparatory materials. Most accessed the content within the intended timeframe, typically 24 to 48 h before class, indicating alignment with the instructional design. The majority of students completed the assigned modules and spent sustained time on narrated presentations and interactive H5P activities (Table 5). Performance on embedded quizzes suggested that students were able to assimilate core concepts effectively before attending face-to-face sessions.

These findings confirmed that students were actively engaging with the theoretical content in advance, fulfilling the foundational layer of the flipped model.

### 3.2. Attendance and Video Engagement Patterns

In 2023/24, asynchronous video visualization surpassed in-person attendance, suggesting that students gravitated towards flexible study habits that supported autonomy and schedule management. However, this preference may also have reflected the absence of structured accountability mechanisms in the design of that year. Conversely, in 2024/25, a more balanced dynamic emerged, with in-person attendance surpassing video views in several sessions. This shift is likely due to the introduction of continuous assessment, whereby students were required to complete graded cognitive exercises in class, thereby reinforcing the importance of attendance and participation. Notably, both academic years showed significant drops in attendance around exam periods for other subjects. However, the behavioral responses differed: in 2023/24, students replaced class attendance with video viewing during peak exam times, whereas in 2024/25, they continued to prioritize attendance. This distinction suggests a stronger perception of instructional value when face-to-face sessions are relevant to assessments—a design choice that enhances motivation and reduces disengagement during academically stressful periods.

### 3.3. Student Engagement Patterns Across Sessions

Student engagement was analyzed by comparing video access before class and in-class attendance across respiratory and cardiovascular sessions in both academic years. The figures below show how students interacted with the flipped content and how their behavior evolved over time.

During the 2023/24 academic year, students typically accessed the H5P^®^ preparatory videos within the allocated five-day period before class. Attendance rates varied across sessions, with respiratory modules showing slightly higher participation than cardiovascular ones. Interestingly, several CARD sessions showed a difference between video access and attendance, suggesting that some students relied on digital preparation instead of attending the corresponding face-to-face sessions.

Similar patterns were observed in 2024/25, but with a notable shift: video access remained high, and attendance improved in sessions where continuous assessment was introduced. This suggests that structured evaluation mechanisms may enhance alignment between preparation and participation.

**Figure 1:**
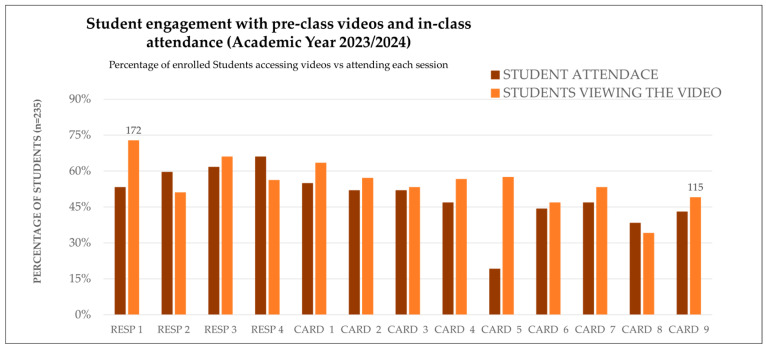
Student engagement with pre-class videos and in-class attendance (academic year 2023/24). This compares the percentage of enrolled students (n = 235) who accessed H5P pre-class videos within the five-day window, with the percentage of students who attended the corresponding sessions. These sessions include respiratory (RESP 1–4) and cardiovascular (CARD 1–9) topics. CARD: Cardiovascular system. RESP: respiratory system.

**Figure 2:**
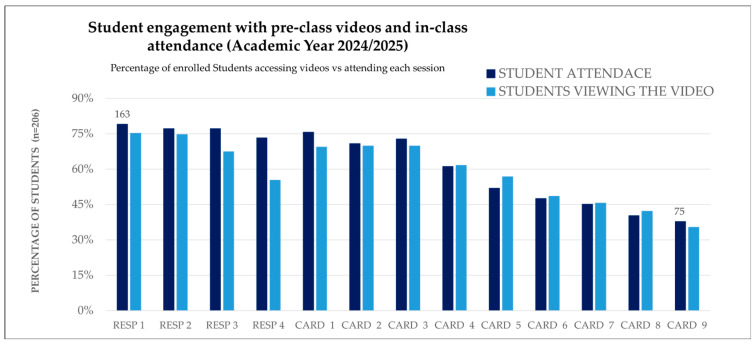
Student engagement across RESP and CARD sessions (2024/25): Comparison of in-class attendance and pre-class video access (H5P). Percentages are based on the total number of students enrolled (n = 208). CARD, Cardiovascular system. RESP, Respiratory system.

Figure 1 and Figure 2 present these comparisons for each session, highlighting the relationship between digital engagement and physical attendance.

Further analysis focused on students who attended sessions without prior video viewing. In 2023/24, this behavior was particularly evident in CARD 3 and CARD 6, suggesting a tendency towards passive attendance. Similar patterns emerged in complex cardiovascular sessions in 2024/25, suggesting that students may have postponed preparation in favor of in-class clarification.

**Figure 3:**
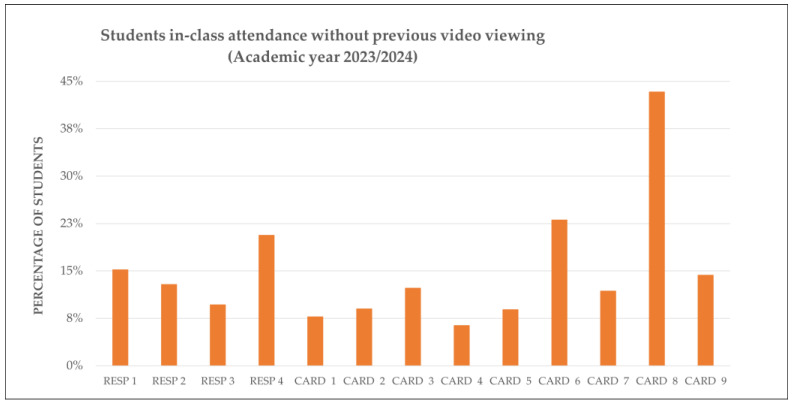
Class attendance without prior video viewing: Academic Year 2023/24. Percentage of students (n = 235) who attended each RESP and CARD session without accessing the corresponding H5P video. CARD, Cardiovascular system. RESP, Respiratory system.

**Figure 4:**
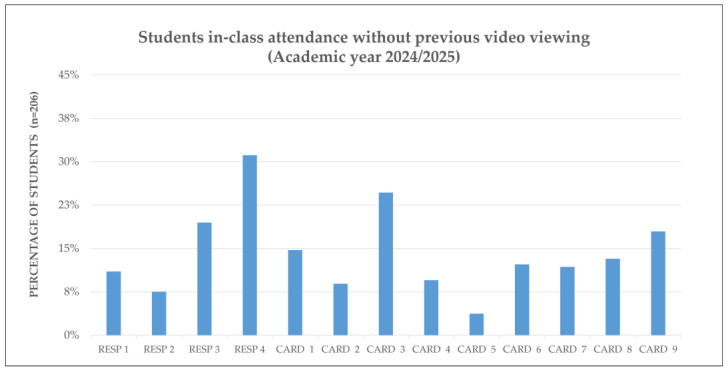
Class attendance without prior video viewing: Academic Year 2024/25. Percentage of students (n = 208) who attended each RESP and CARD session without accessing the corresponding H5P video. CARD, Cardiovascular system. RESP, Respiratory system.

Figure 3 and Figure 4 illustrate the proportion of students who attended class without engaging with the preparatory content, emphasizing the need for clearer guidance and better integration of pre-class materials.

**Figure 5:**
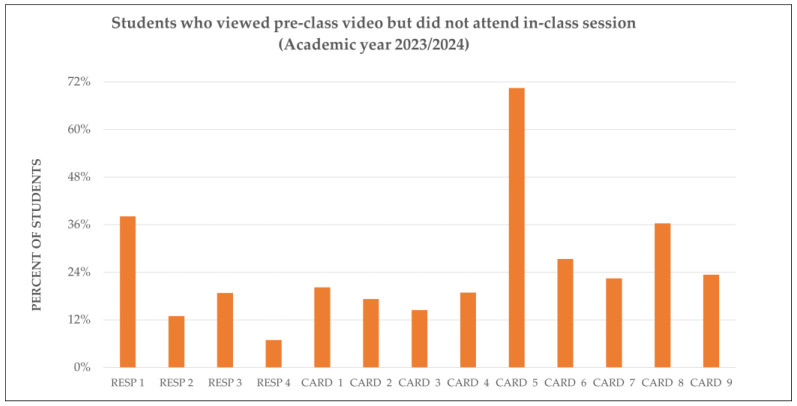
Students viewing flipped content but not attending class (2023/24). This shows the proportion of students who watched H5P videos but did not attend the corresponding RESP and CARD sessions. CARD, Cardiovascular system. RESP, Respiratory system.

**Figure 6:**
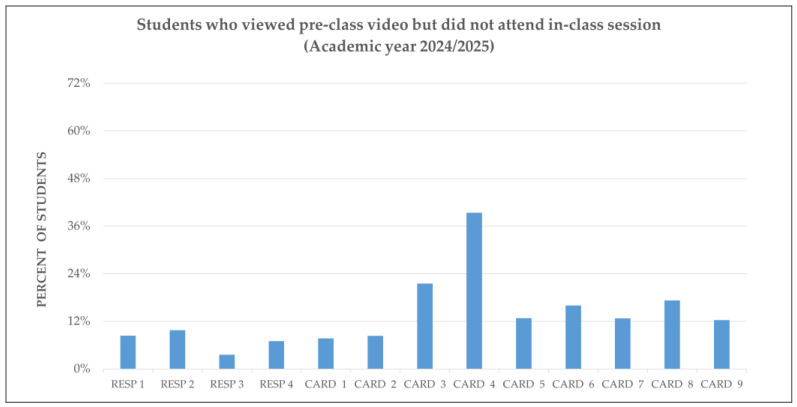
Students viewing flipped content without attending class (2024/25). This shows the proportion of students who watched H5P videos but did not attend the corresponding RESP and CARD sessions. CARD, Cardiovascular system. RESP, Respiratory system.

Finally, Figure 5 and Figure 6 compare the performance of students who viewed the flipped content but did not attend class. In 2023/24, a significant number of students chose to engage with the content solely via video, which undermined the collaborative nature of the flipped model. This behavior declined significantly in 2024/25, coinciding with the introduction of continuous assessment and structured in-class activities.

These findings emphasize the importance of instructional coherence and assessment design in encouraging balanced participation in both elements of the FC.

This bar chart shows the proportion of students who watched the FC videos but did not attend the corresponding live sessions for the RESP and CARD modules.

Figure 5 and Figure 6 show a clear change in student behavior regarding the use of flipped content without attending class between the 2023/24 and 2024/25 academic years. In 2023/24 (Figure 5), a significant proportion of students opted to watch the videos but did not attend the subsequent face-to-face sessions. This suggests that, without continuous assessment or structured incentives, some students perceived watching the videos as an adequate substitute for participating in class. Such behavior undermines the pedagogical coherence of the flipped model, which depends on integrating pre-class preparation with active, collaborative in-class learning.

By contrast, in 2024/25 (Figure 6), the proportion of students who viewed content without attending class was significantly lower. This change coincided with the introduction of continuous assessment and structured in-class activities, which appear to have encouraged greater alignment between preparation and attendance. The data suggest that, when accountability mechanisms are in place, students are more likely to engage with both components of the flipped structure—pre-class study and in-class application—thereby reinforcing the intended learning cycle.

Together, these figures emphasize the importance of assessment design and instructional coherence in sustaining student engagement. While flipped videos provide flexibility, they only achieve their full pedagogical potential when paired with structured, meaningful in-class participation.

### 3.4. Preparation Timing and Cognitive Performance

Engagement data from the H5P platform offers deeper insight into how the timing of video access correlates with cognitive performance. Students were grouped into three preparation categories: viewing 1 day before class, viewing 2–3 days prior, and viewing 4 or more days ahead. For cognitive levels 1 and 2, earlier preparation directly improved performance, consistent with the principles of spaced repetition and memory consolidation. However, at cognitive level 3, performance gains were more uniformly distributed across the 2–3 day and 4+ day ranges, suggesting that analytical tasks rely less on preparation time and more on the ability to transfer and interpret knowledge.

**Figure 7:**
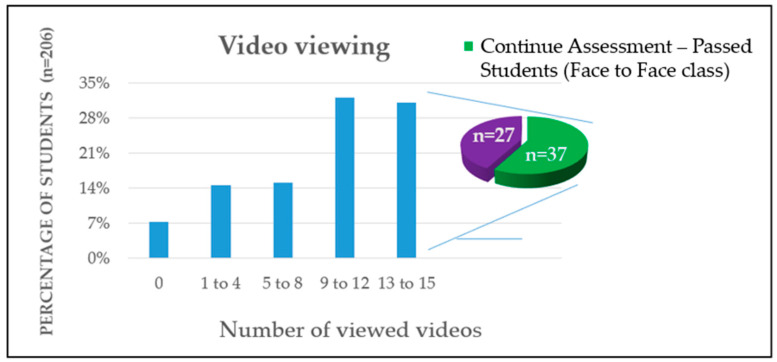
Number of Videos Viewed by Students in Academic Year 2024/25, (including those who passed continuous assessment). The bar chart displays the distribution of students based on the number of FC videos viewed, categorized into five viewing ranges. The majority of students watched between 9 and 15 videos, with each of the top two categories (9–12 and 13–15 videos) comprising approximately 30% of the cohort. The pie chart illustrates assessment outcomes, showing that a substantial portion of students who continued with the assessment successfully passed the face-to-face class.

Figure 7 shows that the majority of students watched a significant number of FC videos, with almost two-thirds of the cohort viewing between nine and 15 sessions. This suggests consistent engagement with the preparatory materials throughout the course. The accompanying pie chart shows that a large proportion of students who completed the assessment successfully passed the face-to-face class, which reinforces the link between sustained engagement with FC resources and positive academic outcomes. Together, these results emphasize the importance of regular interaction with preparatory content as a basis for participation and achievement in class.

Table 6 compares the timing of student engagement with preparatory video materials across two academic years and by topic (RESP and CARD). In 2023/24, the majority of students accessed videos within one day of the class, particularly for CARD sessions (38.83%), suggesting last-minute preparation. In contrast, the 2024/25 data show a shift towards earlier engagement, with increased access in the 2–5 day range, especially in CARD sessions. Same-day viewing dropped to 27.97%, while access 2–3 days prior increased to 12.74%.

### 3.5. Procrastination Trends Across Cohorts

Student procrastination was monitored across two academic cohorts using two key curricular checkpoints: the respiratory system unit, which marked students’ initial exposure to the flipped model, and the cardiovascular system unit, which occurred after students had gained experience with the approach. An analysis of student behavior across the 2023/24 and 2024/25 academic years revealed distinct procrastination patterns linked to instructional design. For example, in the cardiovascular unit, 33% of students viewed preparatory videos the day before class in 2023/24, compared to 21.69% in 2024/25. Furthermore, procrastination increased over time within the first cohort, rising from 29.87% during the respiratory unit to 47.99% during the cardiovascular unit, suggesting that delayed engagement became normalized in the absence of continuous assessment. In contrast, initial procrastination rates were higher in 2024/25 (51.5%) but declined sharply to 18.28% as the course progressed. This suggests that regular evaluative reinforcement and familiarity with the FC model helped to reshape study habits, encouraging more proactive learning.

During the 2023/24 academic year, flipped instruction was introduced without continuous evaluation during in-person sessions, potentially impacting engagement habits. By the time students reached the cardiovascular unit, 33% of them had watched the preparatory videos on the day before the class session, reflecting a tendency to postpone engagement until the last moment. This figure was significantly higher than the 21.69% observed in the 2024/25 cohort, who had continuous evaluation integrated into face-to-face teaching, suggesting that regular accountability fosters more timely participation.

Trends in procrastination over time within each academic cycle further illuminate behavioral shifts. In 2023/24, the percentage of students demonstrating procrastination behavior increased substantially, rising from 29.87% during the respiratory unit to 47.99% during the cardiovascular unit. This suggests that, in the absence of sustained evaluative reinforcement, procrastination became increasingly normalized. By contrast, in 2024/25, 51.5% of students initially procrastinated, but this figure dramatically declined to 18.28% as the course progressed, indicating a reverse pattern in which familiarity with the flipped model, coupled with continuous feedback, may have helped to reshape study habits.

### 3.6. Correlation Between Preparation Time and Cognitive Performance

Student performance was analyzed across four cognitive levels (recall, comprehension, application and integration) to evaluate the impact of preparation time on learning outcomes. These levels aligned with the module’s learning objectives, which emphasized anatomical identification, spatial reasoning, and clinical application. The analysis focused on students who passed the continuous assessment during the 2024/25 academic year.

Overall, the results revealed a positive correlation between earlier preparation and higher performance, particularly at the higher cognitive levels. Students who engaged with the materials two to three days before class consistently achieved better results, indicating that the flipped classroom model encourages deeper cognitive engagement and the development of transferable skills.

**Figure 8:**
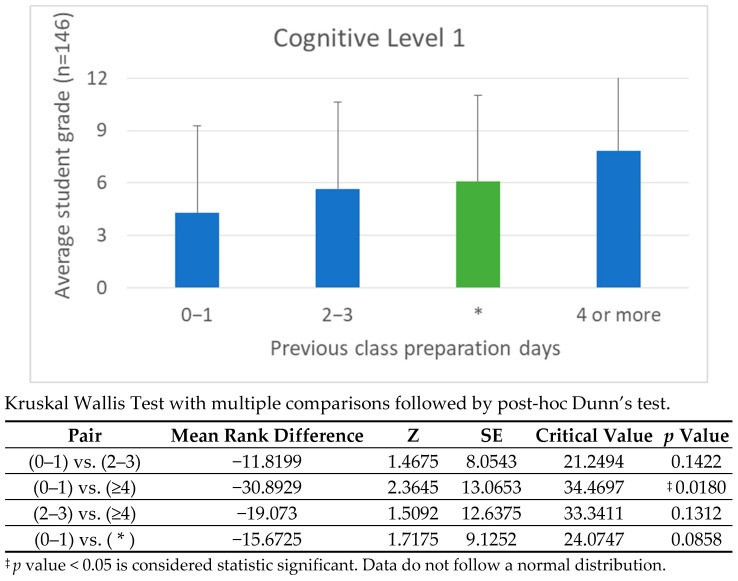
Correlation Between Preparation Time and Performance on Cognitive Level 1 Questions: Academic Year 2024/25. * Students who passed the continuous assessment. This bar chart shows how the number of days that student spent preparing for class correlates with their average grade. At Level 1 (recall), students who spread their study time over several days demonstrated better memory retention. This pattern indicates that factual knowledge is more effectively consolidated when exposure occurs early and is reinforced over time.

**Figure 9:**
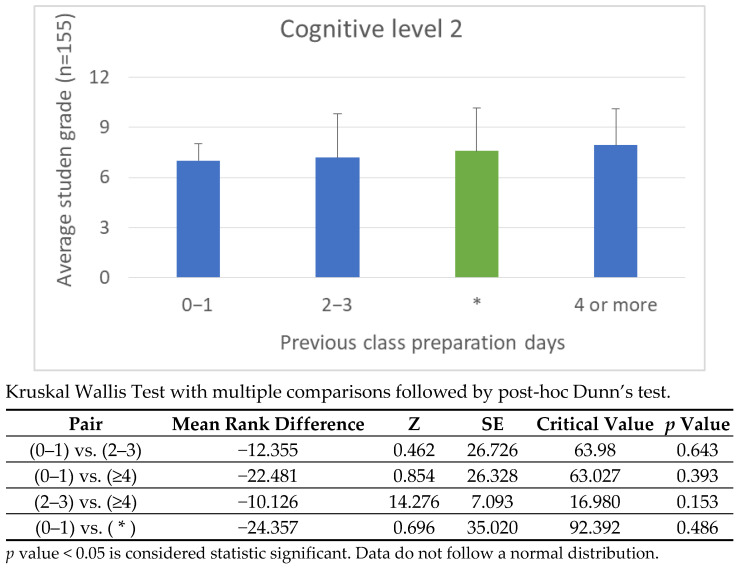
Correlation Between Preparation Time and Performance on Cognitive Level 2 Questions: Academic Year 2024/25. * Students who passed the continuous assessment. This bar graph illustrates the relationship between how many days students spent preparing for class and their average grade at Cognitive Level 2. At comprehension level (level 2), a similar but less pronounced trend was observed. Students who began preparing earlier achieved slightly higher scores, suggesting that more time allows for a more thorough understanding of anatomical concepts to be achieved.

**Figure 10:**
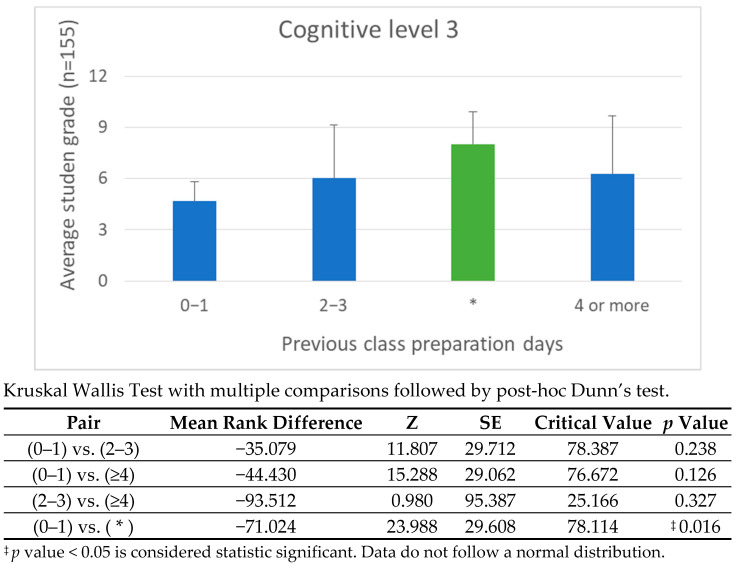
Correlation Between Preparation Time and Performance on Cognitive Level 3 Questions: Academic Year 2024/25. * Students who passed the continuous assessment. This bar graph illustrates how the number of days students prepared before class correlates with their performance at Cognitive Level 3. At the application level (Level 3), the correlation between preparation time and performance became more evident. Students who began preparing at least two days in advance performed significantly better, reflecting the cognitive demands of applying anatomical knowledge to clinical scenarios.

**Figure 11:**
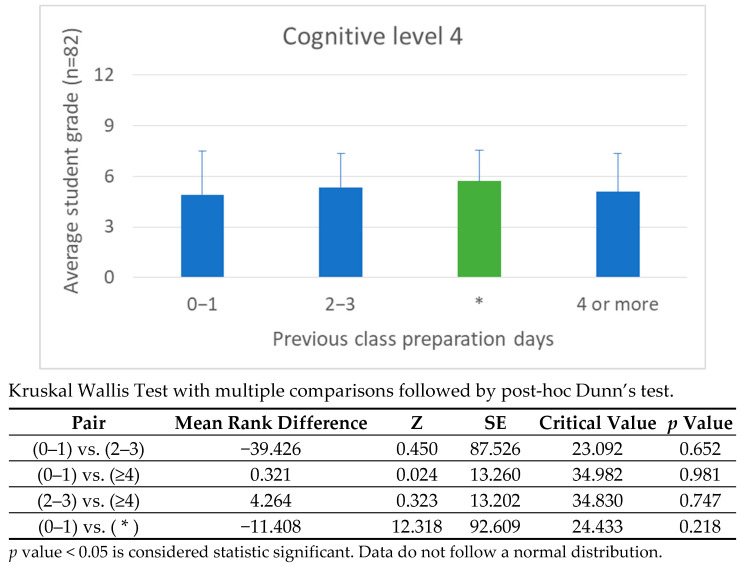
Correlation Between Preparation Time and Performance on Cognitive Level 4 Questions: Academic Year 2024/25. * Students who passed the continuous assessment. This bar graph illustrates the relationship between how many days students spent preparing for class and their average grade at Cognitive Level 4. At Level 4 (the integration level), early preparation was particularly important. Students who began studying two to three days before class achieved the highest scores, indicating that advanced reasoning and synthesis benefit from a balance between recall and reflection.

Figure 8, Figure 9, Figure 10 and Figure 11 illustrate these findings in detail, showing how preparation time influenced performance at each cognitive level. Figure 8: shows the correlation between preparation time and performance on Cognitive Level 1 (Recall) questions. Academic Year 2024/25. Students who passed the continuous assessment. Figure 9: Correlation between preparation time and performance on Cognitive Level 2 (Comprehension) questions. Academic year: 2024/25. Students who passed the continuous assessment. Figure 10: Correlation between preparation time and performance on Cognitive Level 3 (Application) questions. Academic year: 2024/25. Students who passed the continuous assessment. Figure 11: Correlation between preparation time and performance on Cognitive Level 4 (Integration) questions. Academic year: 2024/25. Students who passed the continuous assessment.

These results confirm that, while early preparation benefits all levels of learning, its importance increases as cognitive demands rise. The flipped classroom model fosters deeper processing,

### 3.7. Student Study Behaviors and Perceptions

To complement the quantitative analysis, anonymous surveys were conducted at the end of the module to gather qualitative insights into students’ study habits. The surveys focused on two key aspects: (1) how much time students dedicated to preparing for flipped videos and (2) how they utilized the additional study time afforded by the flipped format. This information is shown in Table 7.

The responses revealed a variety of strategies. While some students used the extra time to review anatomical diagrams or practice clinical reasoning, others prioritized collaborative study or revisited lecture content for reinforcement. These findings offer a nuanced view of how students adapt their learning behaviors when given flexible access to preparatory materials.

### 3.8. Survey Results on Study Habits and Preferences

End-of-module surveys revealed significant changes in students’ study behaviors over the two academic years. In 2023/24, almost half of respondents said they spent only the duration of the video on preparation. By contrast, in 2024/25, the majority dedicated 30 min or more to studying before class, indicating increased effort and engagement. The proportion of students who watched videos for the same length of time as they were long dropped from 48% to 9%, suggesting a shift towards more deliberate and sustained study habits. There was also a significant change in note-taking behaviors: in 2024/25, 67% of students reported taking structured notes during video viewing, reflecting a move towards more active learning strategies. Additionally, students reported reduced reliance on rewatching videos, further supporting the adoption of more intentional preparation practices.

## 4. Discussion

### 4.1. Reframing Theoretical Instruction

This study emphasizes the educational advantages of employing a FC approach to instruct theoretical veterinary anatomy, particularly within the respiratory and cardiovascular modules. By shifting foundational content to independent study supported by H5P^®^ interactive videos and annotated diagrams, face-to-face sessions were repurposed for collaborative problem-solving and clinical reasoning.

Traditional anatomy instruction often relied on passive lectures, which limited engagement and integrative thinking. The FC model addressed this issue by enabling students to explore anatomical terminology, spatial relationships and physiological principles at their own pace, with embedded formative assessments providing reinforcement. In-class activities then built on this foundation through guided discussions and case-based scenarios, fostering deeper anatomical reasoning. Unlike rigid FC models, our approach permitted flexible entry points. For instance, during the CARD 8 session, students meaningfully engaged in problem-based learning (PBL) tasks even though they had not viewed the preparatory video. This suggests that motivation and contextual relevance can activate prior knowledge, supporting adaptive scaffolding over strict sequencing—an approach that is consistent with constructivist principles [24,25,26,27].

Effective FC implementation also depends on accurately estimating and communicating the time required for independent study. Aligning the pre-class workload with the complexity of the in-class tasks proved to be a sound pedagogical approach. The observed correlation between preparation time and performance in higher-order cognitive tasks (see Figure 8, Figure 9, Figure 10 and Figure 11) highlights the importance of careful instructional planning.

Our comparative analysis of the 2023/24 and 2024/25 cohorts revealed that integrating continuous assessment, expanding problem-based learning (PBL) activities and formalizing preparation schedules led to measurable improvements in autonomy, engagement and cognitive performance (see Figure 8, Figure 9, Figure 10 and Figure 11). While this study does not include a direct comparison with traditional lecture-based cohorts, our team’s previous research has addressed this issue [21,23], demonstrating significantly better outcomes among students taught using FC methodologies.

### 4.2. Linking Digital Preparation to In-Class Application

H5P^®^ modules played a central role in the success of the FC approach. They offered visual and auditory explanations of thoracic anatomy, as well as interactive elements such as labeling exercises, branching scenarios and embedded quizzes. These resources were closely aligned with the course objectives, helping students to arrive in class with a coherent conceptual framework. In-class activities were intentionally designed to build on this foundation. After studying pulmonary circulation, for example, students collaboratively mapped blood flow and discussed anatomical variations. Similarly, after watching a video on mediastinal compartments, they explored the clinical implications of space-occupying lesions. These tasks exemplify the FC principle of using classroom time for higher-order thinking and contextual integration.

While the FC model promotes competency development and lifelong learning, its success depends on careful planning and the integration of technology [4]. A key behavioral challenge is student procrastination, particularly in asynchronous settings. Marshall et al. (2022) found that delayed engagement with pre-session materials reduced participation in active learning [14]. Without regular check-ins or graded incentives, students often postpone preparation, repeating habits from traditional lecture-based models [28,29]. Whether this reflects strategic flexibility or poor time management remains a matter of debate. Integrating interactive H5P^®^ activities with clear expectations and structured assessments can mitigate procrastination and foster consistent learning routines. Beyond their immediate instructional value, H5P^®^ modules also serve as enduring digital resources that support ongoing learning [30].

### 4.3. Student Engagement and Pedagogical Coherence

Platform analytics revealed high engagement with pre-class materials, with completion rates exceeding 90% and quiz scores indicating a good level of comprehension. Student feedback confirmed an increase in confidence regarding anatomical knowledge and an appreciation of collaborative learning opportunities. The FC model’s pedagogical coherence was evident in the transition students made from passive reception to active participation, which fostered critical thinking, anatomical literacy and professional communication skills.

The introduction of continuous assessment in 2024/25 encouraged students to study more regularly. Engaging with flipped content earlier on facilitated deeper cognitive processing and better alignment with in-class activities. Students responded to the revised instructional design by adjusting their preparation strategies, particularly for cardiovascular modules where the complexity of the content demanded advance study.

Level 4 tasks yielded the highest scores among students who accessed content two to three days before class, suggesting that strategic timing rather than total study duration optimizes learning. These findings support instructional sequences that balance flexibility with structured expectations, particularly in anatomy education, where spatial reasoning and conceptual clarity are paramount.

Further analysis of the survey data revealed a significant shift in study habits. While in 2023/24, 48% of students limited their preparation time to the duration of the video, by 2024/25, this figure had dropped to 9%, with 85% now dedicating 30 min or more to studying. The proportion of students using structured note-taking increased from 50% to 67%, with more students using their notes to consolidate their understanding rather than rewatching videos. These behaviors reflect a transition from passive to active cognitive processing, which is known to enhance retention and comprehension [31].

Students also reported the high perceived value of flipped materials for practical preparation and the motivational impact of active formats. They also requested stronger integration between pre-class content and practical challenges. These findings are consistent with previous research indicating that meaningful tasks and accessible resources promote autonomy and deeper engagement [32,33,34]. The CARD 8 session further demonstrated that interest and contextual relevance can activate learning even without prior video viewing [35,36].

### 4.4. Implications for Anatomy Teaching and Instructional Design

The FC model enabled students to independently engage with core anatomical content, thereby freeing up classroom time for active learning strategies such as problem-based learning (PBL) and team-based learning (TBL) [4,11,20,21,23,37,38,39,40]. This structure was particularly effective for ILOs involving spatial reasoning and clinical contextualization, as these benefit from peer interaction and guided facilitation.

The intervention was aligned with Marzano’s framework for effective teaching, incorporating goal setting, formative feedback, non-linguistic representation and cooperative learning. Pre-class modules featured interactive diagrams and quizzes, while in-class sessions emphasized clinical application through peer discussion and problem solving. This approach reinforced outcomes such as anatomical identification, functional reasoning and spatial orientation, while promoting self-regulated learning and critical thinking.

Intentional study strategies, such as guided handwriting, were particularly effective in organizing, summarizing and reframing content [31]. This deliberate approach fosters reflection and transforms note-taking into a strategic learning tool.

Student attitudes towards flipped learning also evolved. Initially, many favored traditional lectures and were uncomfortable with independent study [16]. By the end of the course, however, half had embraced the flipped model, while the rest had retained their conventional preferences. These findings echo those of previous studies [14,29] and emphasize the importance of gradual adaptation and pedagogical support.

However, several limitations should be noted. Firstly, the study focused on first-year veterinary students, and prior academic performance was not formally compared. The analysis relied on descriptive and formative indicators, and the findings are specific to a single institution, which may limit their generalizability.

Institutional dynamics also pose challenges to sustainability. Despite the increased demand for instructional support, staffing levels have been reduced. Resistance to pedagogical innovation among colleagues further complicates implementation. In contexts such as the UCM Docentia program, where teaching quality is often assessed through student satisfaction scores, the FC model’s emphasis on individual effort may be seen as less effective. These challenges highlight the need for institutional commitment to educational reform. Future research should incorporate standardized metrics, longitudinal tracking and critical analysis of the structural and cultural factors affecting the viability of flipped methodologies.

## 5. Conclusions

This study demonstrates that a well-designed FC model can significantly improve student engagement and preparation habits, as well as enhancing cognitive performance in veterinary anatomy education. By integrating continuous assessment and interactive tools such as H5P^®^ and Wooclap^®^, students progressed through Marzano’s cognitive levels—from basic retrieval to advanced integration—while developing strategic learning behaviors. Survey data and platform analytics revealed a clear shift from passive video viewing to active study practices, such as structured note-taking and timely preparation. Students responded differently to varying degrees of content complexity, with higher levels of engagement observed in sessions linked to assessment and clinical relevance. These findings emphasize the importance of instructional coherence, adaptive scaffolding and explicit guidance in anatomy teaching. The FC model also fosters a constructivist learning environment that promotes autonomy, motivation and deeper cognitive processing. However, the sustainability of the model depends on institutional support, adequate staffing, and openness to pedagogical innovation. Limitations include cohort specificity and the absence of comparative performance data. Future research should incorporate standardized metrics, longitudinal tracking and critical analysis of the structural and cultural factors that influence the viability of flipped methodologies. When implemented with clarity, consistency and cognitive alignment, the FC model provides a robust framework for contemporary medical education, fostering lifelong learning, clinical reasoning and professional competence.

## Figures and Tables

**Table 1:** Assessment Activities and Their Role.

Activity Type	Description	Role in the Study
Real-time polling (Wooclap^®^)	Spot polls during class to assess group consensus and misconceptions	Immediate feedback and engagement tracking
Problem-based anatomical reasoning	Diagnostic image analysis mapped to anatomical structures	Application of knowledge to clinical scenarios
Reinforcement of theory	Sessions combining function and anatomical landmark identification	Integration of cognitive and practical skills

**Table 2:** Targeted Learning Objectives.

Objective	Role in the Study
Identification of thoracic anatomical structures	Foundation for anatomical recognition
Understanding spatial and functional integration	Supports physiological and anatomical reasoning
Application to clinical scenarios	Bridges theory with diagnostic practice
Development of structured study habits	Encourages independent learning and preparation
Collaborative reasoning in face-to-face sessions	Promote teamwork and peer learning

**Table 3:** Intended Learning Outcomes and Their Functions.

ILO Code	Description	Function in Study
ILO1	Identification and description of thoracic structures and their relationships	Supports anatomical precision and spatial reasoning
ILO2	Explanation of respiratory and cardiovascular interactions	Reinforces physiological integration
ILO3	Anatomical interpretation of clinical scenarios	Develops diagnostic anatomical reasoning
ILO4	Group collaboration and communication	Enhances peer learning and terminology use

**Table 4:** Behavioral Indicators and Data Collection Tools.

Indicator	Description	Role in Study
Baseline and postintervention surveys	Captured initial and final perceptions of students	Qualitative analysis of engagement and perception
H5P module access	Tracked views, timing, and completion rates	Indirect measure of preparation and study habits
Video segmentation	Two 10–15 min videos per module	Cognitive load management and notetaking support
Wooclap^®^ feedback and attendance	Monitored in-class participation and content usefulness	Formative assessment and engagement tracking
Cognitive exercises	Tasks mapped to four cognitive levels (recall to integration)	Quantitative performance analysis

**Table 5:** Summary of Platform Engagement Metrics.

Metric	Observation
Completion rate	Average of 92% across cohorts
Time-on-task	Sustained engagement with narrated and interactive resources
Quiz performance	Average scores above 80% on embedded pre-class assessments
Access timing	Peak usage observed 24–48 h before in-class sessions

**Table 6:** Comparison of student video access patterns in academic year 2023/24 and 2024/25.

Academic Year		Accessed 0–1 Days Before Class	Accessed 2–3 Days Before Class	Accessed 4–5 Days Before Class	Number of Students
2023/2024	RESP	29.93%	8.06%	12.00%	154
	CARD	38.83%	6.76%	4.41%	123
	TOTAL	36.09%	7.16%	6.74%	132
2024/2025	RESP	31.77%	9.73%	8.50%	141
	CARD	27.97%	12.74%	9.30%	114
	TOTAL	29.14%	11.81%	9.05%	122

CARD, Cardiovascular system. RESP, Respiratory system.

**Table 7:** Student Engagement with Flipped Videos: Preparation Time and Study Strategies.

Category	Engagement Type	2023/24	2024/25
Preparation Time for Flipped Videos	Same duration as video	48%	9%
	Video + 10 min	29%	18%
	Video + 20 min	11%	25%
	Video + 30 min	11%	85%
Use of Extra Study Time	Re-watching the video	34%	17%
	Watching multiple times	13%	17%
	Taking notes	50%	67%
	Researching unresolved questions	22%	15%

## Data Availability

The raw data supporting the conclusions of this article will be made available by the authors on request.

## Abbreviations

The following abbreviations are used in this manuscript:

CARD	Cardiovascular System
ICT	Information and Communication Technology
ILO	Intended Learning Outcomes
FC	Flipped Classroom
PBL	Problem Based Learning
RESP	Respiratory System
TBL	Team Based Learning
